# Matrix viscoplasticity and its shielding by active mechanics in microtissue models: experiments and mathematical modeling

**DOI:** 10.1038/srep33919

**Published:** 2016-09-27

**Authors:** Alan S. Liu, Hailong Wang, Craig R. Copeland, Christopher S. Chen, Vivek B. Shenoy, Daniel H. Reich

**Affiliations:** 1 Department of Physics and Astronomy, The Johns Hopkins University, Baltimore, MD 21218, USA; 2 Department of Materials Science and Engineering, University of Pennsylvania, Philadelphia, PA 19104, USA; 3 Department of Modern Mechanics, CAS Key Laboratory of Mechanical Behavior and Design of Materials, University of Science and Technology of China, Hefei, Anhui 230027, China; 4 Department of Biomedical Engineering, Boston University, Boston, MA 02215, USA.

## Abstract

The biomechanical behavior of tissues under mechanical stimulation is critically important to physiological function. We report a combined experimental and modeling study of bioengineered 3D smooth muscle microtissues that reveals a previously unappreciated interaction between active cell mechanics and the viscoplastic properties of the extracellular matrix. The microtissues’ response to stretch/unstretch actuations, as probed by microcantilever force sensors, was dominated by cellular actomyosin dynamics. However, cell lysis revealed a viscoplastic response of the underlying model collagen/fibrin matrix. A model coupling Hill-type actomyosin dynamics with a plastic perfectly viscoplastic description of the matrix quantitatively accounts for the microtissue dynamics, including notably the cells’ shielding of the matrix plasticity. Stretch measurements of single cells confirmed the active cell dynamics, and were well described by a single-cell version of our model. These results reveal the need for new focus on matrix plasticity and its interactions with active cell mechanics in describing tissue dynamics.

The reciprocal relationship between cells and the surrounding extracellular matrix (ECM) is fundamental to the structure and function of tissues. This interplay affects such diverse processes as matrix remodeling^1^, cell proliferation^2^, and intracellular signaling^3^. Cell-populated collagen gels have long been used as a simple model system that can recapitulate the 3D environment that cells experience *in vivo*^4^. Mechanical testing using excised and engineered tissues has led to the development of many mathematical models that attempt to explain their response to an external stretching force. These models are diverse, and collectively contain a range of physical behaviors, including aspects of viscoelasticity and inelasticity^5^,^6^,^7^,^8^. However, with these models it is difficult to determine the relative contributions of the mechanically active cells and the mechanically passive ECM to these material properties. More significantly, the roles of matrix viscosity and plasticity and their impact on the active mechanics in tissues remain unexplored.

To address the question of the interactions of active cell mechanical processes with matrix visocoplasticity, we combined mechanical measurements of bovine pulmonary artery smooth muscle cells (SMCs) in a controlled 3D environment with single cell measurements. We constructed microtissues using microfabricated devices that consisted of arrayed pairs of microscale cantilever force sensors^9^,^10^. SMCs introduced into the device as part of a collagen/fibrin solution self-assembled into coherent microtissues, and magnetic microspheres incorporated onto the cantilever device enabled application of external forces via an applied magnetic field^11^. The deflection of the microcantilevers reported both the externally applied force and the force generated by the microtissues. In addition, we made complementary measurements of the response of single SMCs to applied stretch using force-sensing micropost arrays (mPADS) constructed on flexible membranes.

We studied the mechanical properties of the microtissues and individual SMCs by measuring dynamic force generation during a stretch-unstretch-observation protocol. The single cell stretching experiments provided a direct measurement of the cellular response, while the microtissue system enabled measurement of the complete model tissue. We also directly measured the mechanical properties of the ECM by application of the stretch protocol following the removal of the active cellular contribution from the microtissue by treatment with Triton X-100, which lysed the cells. Measurements of stress and strain characterized the response of the microtissues, single cells, and ECM.

To determine quantitatively the separate cell and matrix contributions to the microtissues’ mechanical properties, we mathematically modeled the stress-strain data from the microtissues, single cells, and ECM. Our model is robust enough to recapitulate accurately the combined response of both the active cellular components and the passive ECM of the model tissues. Our results revealed a viscoplastic response from the microtissues, and that the cells and matrix contribute different components to the dynamics. The active mechanics of the cells dominated the viscous properties of the microtissues, while the plasticity was largely due to the ECM. Crucially, these experiments show that the cells effectively shield the ECM from plastically yielding when under tension.

## Results

### Experiments on 3D SMC microtissues show viscoplasticity

**Figure 1:**
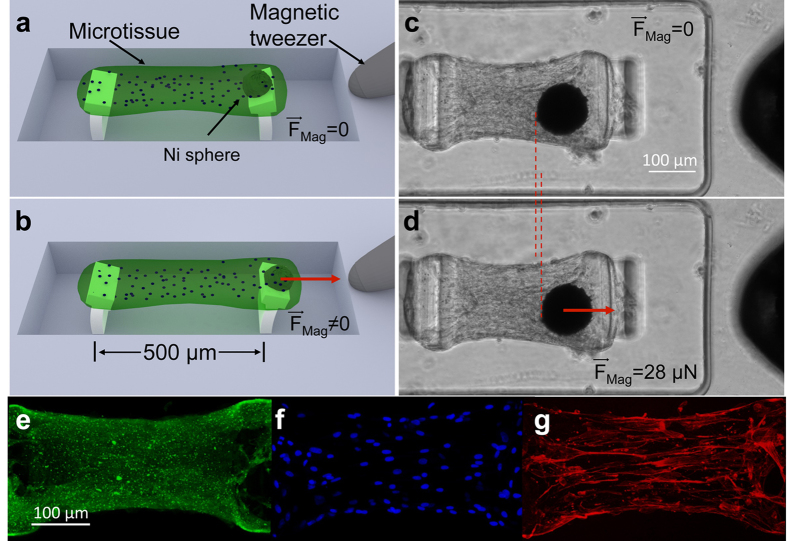
Magnetic microtissue platform for the study of the dynamics of self-assembled smooth muscle tissue constructs. (**a**) Schematic three quarters view showing a microtissue suspended between two flexible PDMS micropillars whose deflections report the microtissue’s contractile force. The wells are 400 μm × 800 μm × 125 μm deep. The flexible lower sections of the pillars were 30 μm × 170 μm in cross section and 85 μm high, and the pillars had spring constants k = 0.59 μN/μm for small lateral deflections. (**b**) A magnetic force **F**_Mag_ applied via a magnetic tweezer to a magnetic Ni sphere bonded to one of the pillars is used to apply time-varying strains to the microtissue. (**c**,**d**) show top-views of a SMC microtissue with (**c**) as grown, and (**d**) subjected to a 2% strain under **F**_Mag._ = 28 μN. The Ni sphere appears as a black circle, and the tip of the magnetic tweezer is visible at the right edge of the images. 2D projected fluorescence confocal images of a microtissue show (**e**) collagen type-I, (**f**) nuclei, and (**g**) F-actin.

To measure the time-dependent biomechanical response of model tissues to external mechanical stimuli, we constructed arrays of engineered microtissues containing bovine pulmonary artery smooth muscle cells (SMCs), as shown in Fig. 1. Such microtissues have been used to measure a range of tissue properties using other cell types^9^,^10^,^11^,^12^,^13^,^14^,^15^,^16^,^17^, but the potential for dynamic studies has not been fully explored. Arrays of microwells were fabricated in polydimethylsiloxane (PDMS). The microwells were 400 μm wide by 800 μm long by 125 μm deep, and each contained a pair of flexible T-shaped PDMS pillars that acted as microcantilevers, as shown in Fig. 1a. A magnetic Ni microsphere bonded to one cantilever in each well enabled application of force^11^,^15^. A collagen/fibrin solution containing SMCs was introduced into the microwells at densities that yielded ~300 cells per well. The contractile action of the SMCs compacted the collagen/fibrin matrix, leading to “dogbone” shaped microtissues suspended between the pillars in each microwell, as shown in Fig. 1c. The microtissues demonstrated a generally uniform distribution of ECM and cells, as shown in Fig. 1e,f, and actin stress fiber bundles aligned predominantly along the long axis of the tissues, as shown in Fig. 1g. As the microtissues formed, an axial stress developed in each tissue due to the collective forces generated by the cells, and the resulting deflections of the PDMS pillars anchoring the microtissues reported these forces. These endogenous forces within the microtissues reached a plateau after approximately 48 hours with average stress in the tissues’ central region 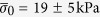 (N = 21) (Supplementary Figs 1 and 2a), after which the mechanical experiments were performed.

**Figure 2:**
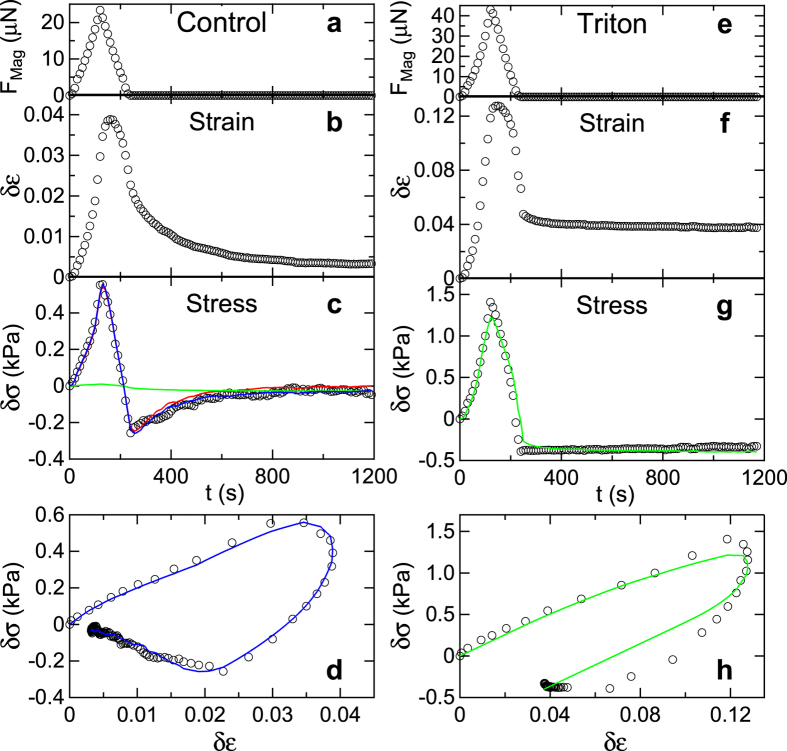
Dynamic response of microtissues to stretch. Shown are the stretch/unstretch responses of an untreated control (Tissue C1) and a Triton X-100 treated microtissue (Tissue T1) to magnetic forcing. (**a**) Magnetic force profile F_Mag_ vs time t applied via Ni sphere to control microtissue. (**b**) Resulting incremental strain *δε* vs t and (**c**) incremental stress *δσ* vs t profiles measured relative to the as-grown tissue configuration, which had a baseline stress *σ*_0_ = 6.66 kPa. (**d**) Incremental stress-strain curve *δσ* vs *δε*. (**e**–**h**) Corresponding results for the Triton-treated microtissue, with *δε* and *δσ* measured relative to the configuration following treatment where *σ*_0_ = 1.56 kPa. The control tissue’s response is characterized by an active recovery to close to its initial state while the Triton-treated tissue shows irreversible plastic deformation. The blue curves in (**c**,**d**) are the result of a fit to the model shown in Fig. 3. The red and green traces in (**c**) show the cell and ECM contributions to the incremental stress, *δσ*_*a*_ and *δσ*_*p*_, respectively, as determined from the model. The green curves in (**g**,**h**) are a fit including ECM contributions of the model only. The parameters for these fits are given in Supplementary Table 1.

Mechanical forces were applied to the microtissues via magnetic forces produced on the nickel spheres by the pole tip of an electromagnetic tweezer, as shown in Fig. 1b,d. We applied a stretch-unstretch protocol in which the magnetic force F_Mag_ was increased to 25–35 μN over 120 s, and then decreased to zero over a similar time interval, as shown in Fig. 2a. The deflections of the pillars caused by F_Mag_ and the motion of the microtissues under test were recorded by time-lapse phase contrast and fluorescence microscopy during force application, and for a 15 min interval after the cessation of the applied force. Figure 2b shows the time evolution of the incremental strain *δε* = *ε* − *ε*_0_ of a microtissue in response to F_Mag_, where *ε* is the absolute strain and *ε*_0_ is the baseline strain before F_Mag_ is applied. Figure 2c depicts the corresponding incremental stress *δσ* = *σ* − *σ*_0_, where *σ* is the absolute stress, and *σ*_0_ is the baseline stress.

The resulting incremental stress-strain loop is shown in Fig. 2d. Both the stress and the strain increased with increasing magnetic force, but while the peak in the stress tracked closely with the peak in F_Mag_, the peak in the strain lagged the peak stress by ~40 s, a hallmark of a viscoelastic response. At the cessation of applied force, at time t = 240 seconds, the microtissues were still in a strained state, while the stress was lower than its initial value. Over the subsequent observation period the tissues recovered to close to their initial states, but maintained a small amount of residual strain. Analogous data for additional microtissues are shown in Supplementary Fig. 3.

### Microtissue plasticity can be attributed to the ECM

The small residual strain observed in the tissues following force application suggested that the dynamic response of the tissues included plastic deformation. This was more clearly evident in the behavior of tissues after treatment with a 0.1% solution of Triton X-100, which lysed the cells and removed any active cellular processes from the force dynamics of the microtissues. The Triton-treated microtissues generally showed similar morphologies to untreated control tissues, but with reduced baseline stress *σ*_0_ as expected from the lysis of the cells (Supplementary Fig. 2a). Figure 2e–h show the response of a Triton-treated microtissue to the stretch-unstretch actuation. The initial response was qualitatively similar to the control tissues, although with reduced initial stiffness, as determined by the initial slope of the stress-strain curves shown in Fig. 2h and Supplementary Fig. 2b. In contrast to the untreated tissues, however, the residual strain following the cessation of applied force was permanent in Triton-treated microtissues, as shown in Fig. 2f, indicating plastic deformation. In addition, Triton-treated microtissues showed no stress recovery, as shown in Fig. 2g and in the corresponding stress-strain loop in Fig. 2h. (See Supplementary Fig. 4 for additional examples.) These results show that the recovery observed in the control tissues was due to active mechanical processes in the cells and, importantly, imply that the observed plasticity resulted primarily from the mechanical response of the ECM, and that this plasticity was largely shielded by the cells in the intact tissues.

### A mathematical model of microtissue mechanics

**Figure 3:**
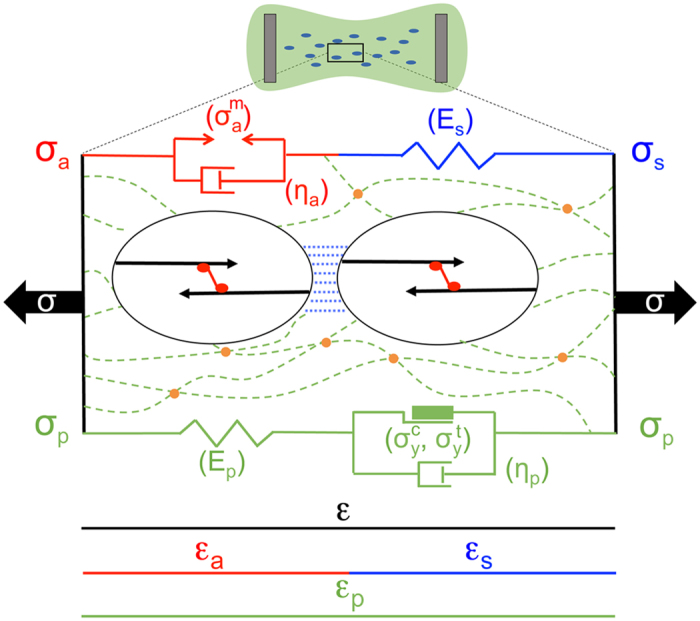
Model for the mechanical response of a microtissue. A microtissue is shown schematically at top, and the model is shown below. The model includes a passive elastic element *E*_*s*_ (blue) that accounts for internal cell stiffness (black), and cell-cell contact stiffness and aligned regions of ECM between cells (dashed blue). *E*_*s*_ is in series with an active cellular contractile element (red) that obeys a Hill-like stress vs. strain-rate relation, with constant maximum active contractile stress 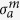 and active viscosity *η*_*a*_. Passive model elements (green) act in parallel to the active elements and represent the behavior of the majority of the ECM, which is under compression in the native state (green dashed lines), and include stiffness *E*_*p*_ and a viscoplastic element with tensile and compressive yield stresses 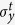 and 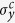, and plastic viscosity *η*_*p*_. *σ* and *ε* denote the total stress and strain of the microtissue, and the subscripts a, s, and, p delineate the stresses and strains of the active, series, and parallel elements, respectively. The quantities indicated in brackets are the material parameters of the model.

To describe the observed behavior and to determine quantitatively the material parameters of our constructs, we developed a three-element model for the biomechanical response to applied force, and used it to fit the data from our tissue and single cell experiments. This model, shown in Fig. 3, builds upon our previous work on active mechanics^17^, but adds additional features to account for the potential effects of ECM plasticity. Our model includes an active contractile element to describe the force generation of the cellular actomyosin system, and a passive elastic element in series with the active element to model the stiffness of the cellular actin filaments and cell-cell contacts, and the stiffness of ECM regions between the cells, which may be aligned and under tension^18^. In parallel with these is an elastic viscoplastic element that models the majority of the ECM, which is compressed as the microtissues form. (The elastic component of this element also accounts for any cellular components that act in parallel to the cellular active element). The reference configuration of the microtissue is after contractile stress activation and the corresponding compaction of the ECM, but before the application of magnetic force. (Compaction is defined as the state where the ECM has yielded under compressive stress). All the elements’ stresses and strains are variables under uniaxial loading. The total strain of the microtissue *ε* is decomposed into active contractile and passive series components, *ε*_*a*_ and *ε*_*s*_. Further, since the viscoplastic element deforms in parallel with the cells, its strain *ε*_*p*_ is equal to the total strain, and thus


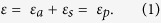


Since the active contractile and passive series elastic cellular elements transmit the same forces, their stresses *σ*_*a*_ and *σ*_*s*_ are the same, and the total stress at any point is the sum of that stress and the stress from the parallel components *σ*_*p*_, so that


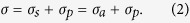


To develop the constitutive laws for each mechanical component needed to relate the stress at any material point to the deformation, we note that all the strains induced by our magnetic field actuation are small, in particular relative to the strain-stiffening threshold seen in collagen and other biological gels^19^,^20^. Further, over this range of strains, we found a strong linear correlation between the peak incremental stress *δσ*_*peak*_ and the strain at *δσ*_*peak*_ for the microtissues (see Supplementary Fig. 5). We therefore use linear elasticity to model the series element, *σ*_*s*_ = *E*_*s*_*ε*_*s*_, and an elastic perfectly viscoplastic solid to model the parallel element (Bingham-Norton model)^21^,


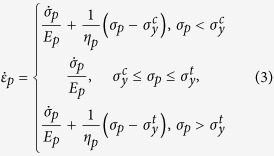


where 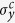 and 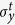 are the compressive and tensile yield stresses, respectively, and *η*_*p*_ is the viscosity of the ECM when the applied stress *σ*_*p*_ is beyond the yield stress range. Both elastic and plastic elements are included in Eq. 3 to account for the fact that the ECM is not ideally plastic, and retains elasticity after yielding (see below). Constant yield stresses are assumed, as other experimental studies have shown that collagen and fibrin strain hardening occurs at strains of greater than 10%^19^,^20^. Recent experiments on fibroblasts subject to uniaxial loading have shown that their active stress vs. strain-rate response obeys the classic Hill relation^22^, and therefore we assume that the active strain rate 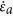 depends on *σ*_*a*_as


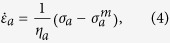


where *η*_*a*_ is the active viscosity, and 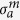 is the constant maximum contractile stress (stall stress) of the cells.

To compare the model to the data, we used the measured incremental strain as input, and fit the computed incremental stress vs. time to the measured incremental stress using custom software that combined numerical solutions to Eqs 1, 2, 3, 4 with a least-squares fitting algorithm. A representative best-fit curve for the fit to the full model for the control tissues is shown in Fig. 2c,d and additional examples are shown in Supplementary Fig. 3. The model parameters determined from these fits are listed in Supplementary Table 1. Overall, the quality of the fits is very good, indicating that the model captures the essence of the tissues’ dynamics.

The Triton-treated tissues were well described by a reduced version of the model that included only the elastic perfectly viscoplastic element (lower branch only in Fig. 3), and omitted the elements describing the cells’ contribution. Figure 2g,h and Supplementary Fig. 4 show results of fits with the reduced model, and the resulting model parameters are given in Supplementary Table 1. The reduced model provides a good description of the observed behavior, including notably the matrix plasticity.

Figure 2c also shows the time courses of the “cellular” and “matrix” components of the stresses *σ*_*a*_ and *σ*_*p*_ as determined by the model. *σ*_*a*_ is significantly larger than *σ*_*p*_, indicating that the cells dominate the active response of the control tissues. We also note that for the untreated control tissues, the model was largely insensitive to the parameters associated with the ECM components, and indeed in many cases we could obtain a good description with a simplified model that included the active and series components of the full model only. Examples of comparisons of the simplified and full models are shown in Supplementary Fig. 6. This insensitivity to the ECM parameters is further indication of the mechanical shielding of the matrix by the cells.

### Cellular active mechanics defines viscosity of microtissues

**Figure 4:**
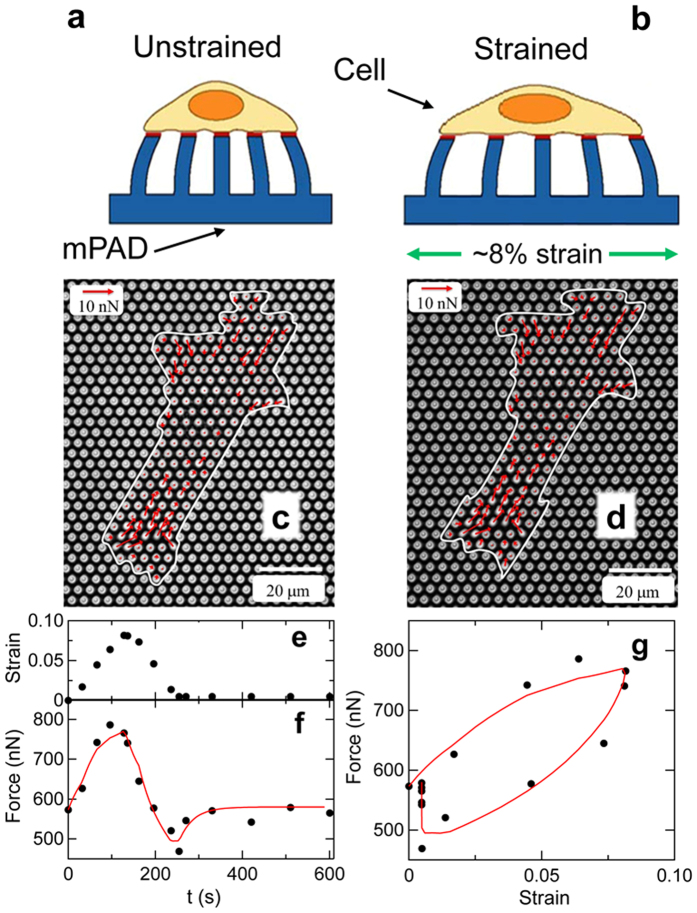
Stretch response of single smooth muscle cells. (**a**) Sideview schematic of cell adhered to micropost substrate (mPAD) in unstretched condition. The magnitude and direction of the deflections of the microposts are used to determine the forces exerted by the cell. (**b**) As the underlying mPAD substrate undergoes biaxial stretch, so too does the adherent cell. (**c**) Micrograph of SMC on an mPAD substrate with an effective micropost spring constant for small lateral deflections of 22 nN/μm. Red arrows show measured cellular forces, and white trace shows the outline of the cell. (**d**) Change in cellular forces upon application of 8% strain. (**e**) Strain profile applied to mPAD substrate results in the increases in cellular force shown by the circles in (**f**). (**g**) Force-strain curve showing viscoelastic response and recovery corresponding to that observed in the microtissues. The red curves in (**f**,**g**) are a fit using solely the cellular components of the model shown in Fig. 3.

To provide an independent assessment of the contribution of the cells to the microtissues’ mechanics, we measured the time-dependent mechanical response of single SMCs to an applied stretch. Micropost array detectors (mPADs)^23^,^24^ constructed on flexible substrates reported the cells’ traction force dynamics as the mPADs were stretched biaxially with time courses analogous to that applied to the microtissues. This stretching is shown schematically in Fig. 4a,b and for a cell in Fig. 4c,d. As illustrated in Fig. 4e, the cells were subjected to a linearly increasing strain reaching a maximum of 10% over 3 minutes, followed by a linear decrease in strain back to the initial state over the same time interval, and then a 15 min strain-free observation period. Figure 4c,d show the increase in traction forces generated by a cell from the unstrained to the maximally strained state. Figure 4f shows the time evolution of the summed magnitudes of the individual forces exerted on the microposts, 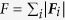, which provides a measure of the overall contractile state of the cell, as the microposts are all bent towards the cell’s center by the cellular traction forces. As shown in Fig. 4g, the response of the total force to applied stretch is qualitatively similar to the stress-strain curves in the microtissue system: at the cessation of stretch, the total cellular force is lower than the initial value, and slowly recovers toward the initial value during the 15 minute observation period following the stretch protocol.

We note that the SMCs adopt polarized configurations on the mPADs, with the traction forces exerted on the microposts oriented primarily parallel to the cell polarization axis 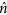. Specifically, we find the ratio 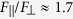, where 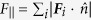 and 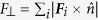 are the summed force magnitudes parallel and perpendicular to 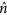, respectively. For all cells measured, upon stretching we observed larger increases in *F*_||_ than in *F*_⊥_, indicating that our quantification of the force changes in the single cell measurements (Fig. 4) is principally sensitive to this primarily uniaxial response, despite the application of biaxial stretch.

The dynamics of the individual cells’ traction forces in response to stretch could be described using a model that contains only active contractile and passive series elements, similar in form to those of cellular elements of our mechanical model for the microtissue (*i.e.* the top branch in Fig. 3). Figure 4f,g show fits to this model for the cell shown in Fig. 4c,d. From such fits we determined the average effective active viscosity of an individual cell to be 
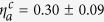
 mN·s. (The units mN·s for 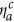 arise because we fit the force magnitude *F* vs. strain in Fig. 4, and not stress vs. strain as in Fig. 2.) There are clearly differences between the geometry of a cell adhered to an mPAD and that of the cells in the microtissues, but notwithstanding, the expected range of the active viscosity of a microtissue 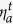 may be estimated from 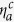, as it should scale with the cross-sectional areal density of cells in the microtissues, *ρ*_*A*_, i.e.


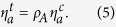


Estimating *ρ*_*A*_ from the cell number density *ρ*_*c*_ 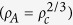, we find that this simple relation gives reasonably close agreement with the values for the active viscosity 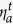 obtained from the fits to the microtissue data (Supplementary Table 1). This, together with the lack of stress recovery in the Triton treated tissues (e.g. Fig. 2), supports the conclusion that the active mechanics of the cells dominates the dynamic response of the microtissues. As additional evidence for the dominance of active cell processes, we note that microtissues formed from cells expressing constitutively active RhoA to upregulate myosin activity^25^ also show significant increase in both baseline stress and initial stiffness (Supplementary Fig. 2).

## Discussion

The microtissue constructs studied here are, like tissues *in vivo*, a composite system consisting of cells and extracellular matrix with properties that are determined by a dynamic interplay between the active cellular mechanics and the complex passive mechanics of the ECM. The experiments described herein have allowed us to decouple the cell and matrix mechanics, and to identify how these subsystems contribute to the tissue’s overall biomechanical response. By measuring the specific contributions of the ECM and the individual cells to the mechanical properties of microtissues in the context of applied stretch, we have revealed a viscoplastic component to the matrix that is largely shielded by the active dynamics of the SMCs. By measuring the time-dependent mechanical response of individual SMCs, we found that the cells’ loading and unloading curves do not coincide but instead show hysteresis, with a force undershoot immediately following cessation of applied strain. Over time, after unloading the SMCs ultimately return to their original state of force generation, as shown in Fig. 4e–g. This is consistent with dynamics reported for other single-cell studies in the literature^26^,^27^, and is consistent with linearized Hill-like dynamics. These cellular dynamics carried over to the microtissues, as shown in Fig. 2.

**Figure 5:**
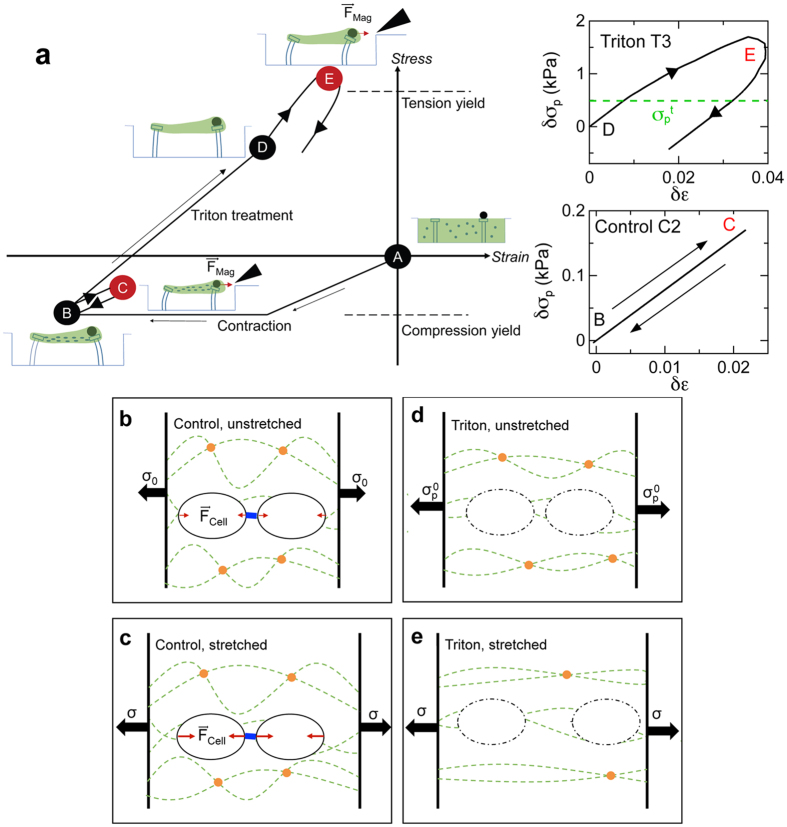
Schematic of matrix mechanics emerging from current studies. (**a**) Stress-strain space diagram for the matrix in the microtissues with schematics showing the relative deflections of the micropillars. Red indicates that the magnetic field is active. The moment of cell seeding is taken to be the zero stress and zero strain condition (Point A). As the microtissue matures, cell contraction compresses the matrix (Point B). Mechanical loading and unloading drives the matrix between points B and C, causing reversible stretching (lower right Inset). Treating microtissues with Triton X-100 lyses the cells placing the matrix under tension due to the outward stress exerted by the micropillars with no counterbalancing active cellular contraction (Point D). Applying the loading profile to Triton-treated microtissues drives the matrix to point E. However, they never fully recover to point D as the tension yield stress 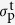 is exceeded (upper right Inset). (**b**–**e**) Illustrations of the state of the ECM during these processes, highlighting the shielding effects of the cells. (**b**) SMCs (black ovals) are dispersed throughout the cross-linked (orange circles) collagen ECM (dashed green lines) and bind with it at focal adhesion sites. The total magnitude of the cellular active contractility F_cell_ is represented by the red arrows. Contractile forces are directed towards the cells’ centers, and the SMCs act as nodes of contractility for the ECM network. Blue bar represents cell-cell mechanical linkages. (**c**) As the tissue is stretched through the application of an external force, tissue stress σ increases as does the levels of cellular force generation. Due to the active contractility of the cells, the collagen network is shielded from plastic deformation caused by this stress increase, and mainly deforms elastically. (**d**) Permeabilizing the cell membrane and lysing the cells through Triton X-100 treatment places the microtissue under tension due to the stress produced by the deflected micropillars (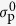). (**e**) Applying an additional external stress σ causes irreversible changes in the ECM microstructure through fiber alignment and changes in crosslinking, leading to plastic deformation.

Due to the shielding of the ECM’s plastic behavior by the cells, accessing the mechanical properties of the ECM required inhibition of the active cellular contribution to the tissues. We achieved this through Triton X-100-induced lysis of the cells. The picture of the matrix mechanics that emerges from these studies is illustrated Fig. 5a, which shows a stress-strain phase space diagram of the matrix component of the microtissues. Here, the zero stress and zero strain condition is taken to be the moment immediately after seeding the cells and matrix in the microwells (Point A). The distributed active contractile forces produced by the cells dispersed in the matrix placed the matrix in the constructs under compression (Point B) as the matrix compacted from its original (seeding) density as the microtissues formed. We note that the stress field inside the microtissues will have some heterogeneity, and despite the overall compression, local regions between cells may even be in tension as they transmit traction forces between cells^18^. This is captured by the element *E*_*s*_ in our model. However, once the active cellular contractility was eliminated via Triton treatment, it became clear that the ECM had in fact yielded under the compressive stress during the tissue formation process. This is evident since, after Triton treatment (Point D), the pillars supporting the tissues remained inwardly deflected, indicating that the microtissues retained residual elasticity that did not depend on the cells. This irreversible change in the matrix during the microtissue formation may be attributed to collagen compaction and subsequent crosslinking as seen in fibroblast constructs^11^,^28^, which is consistent with the recently observed time-dependent inelastic behavior of collagen networks^29^. The compacted, crosslinked state of the as-grown tissues is sketched in Fig. 5b. The mechanical loading and unloading of the tissues during stretch, unstretch, and subsequent active recovery (Path B −>C −>B in Fig. 5a), caused essentially reversible changes in the ECM. This is illustrated pictorially in Fig. 5c, and in the lower inset to Fig. 5a, which shows the matrix component to the total stress-strain response of Tissue C2, as determined from the fit shown in Supplementary Fig. 3. The approximately 10 times smaller scale for the changes in the matrix stress during loading compared to that of the overall stress changes highlights the minimal contribution of the stiffness of the ECM when loaded while under compression.

Upon cell lysis, the removal of the active contraction of the cells shifts the ECM from a state of compression to a state of tension, as the only remaining forces acting on the ECM are those produced at the ends of the tissues by the bending of the micropillars, which attempt to restore the pillars to a vertical orientation. This state of the tissue (Fig. 5d) becomes the new reference stress configuration for the Triton-treated tissues (Point D in Fig. 5a). When a Triton-treated tissue is subjected to an applied stretch (Path D −>E in Fig. 5a), the contractile force of the cells is no longer present to shield the ECM. The ECM now yields under *tension* and undergoes plastic deformation, as illustrated schematically in Fig. 5e, and as shown in the upper inset of Fig. 5a for Tissue T3 (Supplementary Fig. 4). This view of the matrix loading also explains the ostensibly surprising result that larger values of ECM stiffness *E*_*p*_ were observed for the Triton-treated vs. the control tissues. Since the ECM for control and Triton-treated tissues experiences net compressive and tensile stresses, respectively, the ECM stiffness of the Triton-treated tissues is significantly larger than that for the control tissues due to the increased stiffness of gels when under tension as compared to when under compression^30^.

This study has provided a first measurement of the separate contributions of cells and ECM to tissue mechanics that include both the non-linear and plastic properties of the ECM. Observations of the interactions between these two components have revealed that the active mechanics of the cells can shield the ECM from viscoplastic yielding under externally applied stress. Further, the transition of the ECM from a compression-dominated to a tension-dominated regime upon loss of the internal contractile stresses arising from the cells led to an effective stiffening of the ECM. While our model provides a quantitative phenomenological description of the observed plasticity, the mechanism underlying these effects remains an open question. We note that previously, Guidry and Grinnell have suggested that non-covalent crosslinking can occur in collagen gels subjected to compaction and tension^31^. Rearrangement and/or breaking of such linkages could potentially provide an explanation of our yield hypothesis. However, the detailed biochemical processes that could lead to this crosslinking are still unknown, and require further work.

There are potentially significant physiological implications of our results, as such stiffening in response to down-regulation of cellular active mechanics in response to injury or disease could provide a substrate for further pathophysiological changes and exacerbation of disease progression. This current study should also have important implications for future tissue engineering efforts. Previous work has shown that mechanical conditioning of vascular tissue constructs leads to more robust tissue properties^32^. The experimental and mathematical tools described in this work for the *in situ* mechanical conditioning of microtissues have the potential to provide a better fundamental understanding of how the mechanical properties of tissues change as the component cells and ECM develop and mature. This information can provide insight into the effects of tissue engineering parameters on the quality of tissue constructs, particularly in the rapidly growing field of regenerative medicine, where there is a need for experimentally validated constitutive models of tissue behavior.

## Methods

### Cell Culture and Reagents

Bovine pulmonary artery smooth muscle cells (SMCs) (gift from Donald Ingber) were cultured in Dulbecco’s Modified Eagle Medium (Invitrogen) containing 10% bovine serum (Gibco) and 100 units/mL penicillin and 0.1 mg/mL streptomyocin (Invitrogen). The SMCs were cultured at 37 °C in 10% CO_2_ and were passaged 1:4 upon reaching confluence. Cells used in this experiment were at passage 9–16.

### Microtissue Device Fabrication, Seeding, and Imaging

The magnetic microtissue microwell array devices were fabricated as described previously^11^,^15^ from poly(dimethylsiloxane) (PDMS) via replica molding^9^. Each well contained a pair of flexible micropillars whose tops were tagged with 2 μm diameter fluorescent beads (Sigma) to track their deflections. An approximately 100 μm diameter nickel sphere (Alfa Aesar) was attached to one pillar in each well to enable magnetic actuation. To create the microtissues, the arrays were seeded^9^,^11^ with SMCs, 2.5 mg/mL rat tail collagen I (BD Biosciences) and 2 mg/mL fibrinogen from bovine plasma (Sigma)^10^ at a density of 200–400 cells per microwell. The cells compacted the matrix and formed microtissues within 12 hours of seeding. Experiments were done 48 hours after the initial cell seeding. To isolate the ECM contributions to the microtissues’ dynamics, selected microtissues were treated with a 0.1% solution of Triton X-100 for 10 minutes to lyse the cells. Confocal imaging was done on fixed samples stained for collagen type I (AB755P primary antibodies, Millipore; fluorophore-conjugated, anti-IgG secondary antibodies (Invitrogen)), F-actin (Tritc-phalloidin, Sigma), and nuclei (Hoechst 33342, Invitrogen), and 2D projections were obtained by averaging the fluorescent intensity over the confocal stacks.

### Force Application and Stress and Strain Quantification

Forces were applied to individual microtissues by actuating the magnetic pillars via magnetic fields generated by a micromanipulator-mounted electromagnetic tweezer under computer control at 37 °C^11^,^15^,^33^. The micropillars’ deflections were measured via imaging (10x objective, Nikon TE −2000) of the attached fluorescent beads, whose movement was tracked using a custom Matlab script^34^ and the SpotTracker plug-in in ImageJ (NIH). The force on a microtissue during magnetic actuation was determined from the deflection of the non-magnetic pillar. For small deflections, the pillars had a spring constant k = 0.59 μN/μm^9^, and finite element modeling was used to determine the force-deflection curves for larger, non-linear deflections^14^. Tissue strain was quantified using a texture correlation image analysis algorithm applied to the central region of the tissues and from the micropillars’ deflections^11^,^35^.

### Flexible mPAD Devices: Fabrication, Seeding and Application of Strain to Single Cells

Flexible PDMS membranes 300 μm thick were fabricated from 1:20 primer:base ratio PDMS. Micropost array devices (mPADs) were formed on the membranes by replica molding^24^ of 1:10 PDMS. The micropost arrays consisted of 1.8 μm diameter posts, 5.7 μm in height, arranged in hexagonal close-packed arrays, with lattice constant 4 μm. The effective spring constant for the microposts for small deflections was 22 nN/μm. The mPAD membranes were mounted in a custom stretch chamber (Supplementary Fig. 7)^36^ and SMCs were incubated for 24 h on the mPADs to allow the cells to adhere and spread. Biaxial stretch was applied via vacuum actuation of the membrane as controlled by an automated syringe pump.

### Comparison of Model to Data

The materials parameters for each microtissue were obtained by fitting the stress vs. time curves using a random search algorithm^37^, with the strain vs. time curves taken as known input. For each iteration of the search, simulation curves were generated and compared with the experimental data. The optimized parameters were obtained by minimizing the normalized sum of squared residuals *χ*^2^ until *χ*^2^ < 2.5 × 10^−3^ or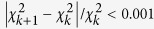, where *k* is the iteration number. An expanded Methods description is given in the Supplementary Information.

## Additional Information

**How to cite this article**: Liu, A. S. *et al*. Matrix viscoplasticity and its shielding by active mechanics in microtissue models: experiments and mathematical modeling. *Sci. Rep.* **6**, 33919; doi: 10.1038/srep33919 (2016).

## Supplementary Material

Supplementary Information
