# Supplementary material for: Matrix viscoplasticity and its shielding by active mechanics in microtissue models: experiments and mathematical modeling
Source: Sci Rep. 2016 Sep 27;6:33919. doi: 10.1038/srep33919 (PMC5037370; doi:10.1038/srep33919)
Supplement: Supplementary Information [file srep33919-s1.pdf]

Supplementary Information for

“Matrix viscoplasticity and its shielding by active mechanics in microtissue models: experiments and mathematical modeling”

Alan S. Liu[a], Hailong Wang[b,c], Craig R. Copeland[a], Christopher S. Chen[d], Vivek B. Shenoy[b,1], and Daniel H. Reich[a,1]

[a]Department of Physics and Astronomy, The Johns Hopkins University, Baltimore, MD 21218; [b]Department of Materials Science and Engineering, University of Pennsylvania, Philadelphia, PA 19104; [c]Department of Modern Mechanics, CAS Key Laboratory of Mechanical Behavior and Design of Materials, University of Science and Technology of China, Hefei, Anhui 230027; [d]Department of Biomedical Engineering, Boston University, Boston, MA 02215.

[1]To whom correspondence should be addressed.
Emails: reich@jhu.edu, vshenoy@seas.upenn.edu.

Expanded Methods:

Microtissue Device Fabrication, Seeding, and Imaging. The magnetic microtissue array devices were fabricated as described previously[1,2]. Briefly, 10 x 13 arrays of 400 μm x 800 μm x 125 μm microwells were fabricated in poly(dimethylsiloxane) (PDMS; Sylgard 184, Dow Corning) substrates via replica molding[3]. Each well contained a pair of flexible, vertical PDMS micropillars separated by 500 μm. The tops of the micropillars were tagged with 2 μm diameter fluorescent beads (Sigma) to track their deflections. An approximately 100μm diameter nickel sphere (Alfa Aesar) was bonded with PDMS to one pillar in each well to enable magnetic actuation.

To create the microtissues, the arrays were sterilized with 70% ethanol and subjected to a 30 min treatment with 0.2% Pluronic F127 (BASF) to reduce cell adhesion, following previously described methods[1,3]. A liquid, chilled ECM solution containing 2.5 mg/mL rat tail collagen I (BD Biosciences) and 2 mg/mL fibrinogen from bovine plasma (Sigma) was added to the substrate[4]. After degassing to remove air bubbles, approximately 3×10^5 SMCs suspended in ECM solution were seeded by centrifugation into the arrays of microwells; this number of cells led to a density of 200-400 cells per microwell. The substrate was then incubated at 37˚ C to induce polymerization, after which media was added. The cells compacted the matrix and formed microtissues within 12 hours of seeding. Experiments were done 48 hours after the initial cell seeding. To isolate the ECM contributions to the microtissues’ dynamics, selected microtissues were treated with a 0.1% solution of Triton X-100 for 10 minutes to lyse the cells.

For confocal imaging, microtissues were fixed with 4% paraformaldehyde in PBS, permeabilized with 0.1% Triton X- 100 in PBS, incubated with antibodies against collagen type I (AB755P, Millipore) and detected with fluorophore-conjugated, isotype-specific, anti-IgG antibodies (Invitrogen). F-actin was stained with Tritc-phalloidin (Sigma), and nuclei by Hoechst 33342 (Invitrogen). Confocal images were obtained on a Zeiss LSM-510 confocal microscope and the confocal stacks were projected to 2D by averaging the fluorescent intensity through all

slices.

Force Application and Stress and Strain Quantification. Forces were applied to individual microtissues by actuating the magnetic pillars via magnetic fields generated by a micromanipulator-mounted electromagnetic tweezer under computer control at 37 °C[1,2,5]. The magnitude of these forces were determined from previously tabulated relations between tweezer current and tweezer-Ni sphere separation and magnetic force[1,2]. To quantify tissue forces, the deflection of the micropillars was measured using fluorescent images taken with a 10x objective (Nikon TE -2000). The tops of the micropillars were tagged with fluorescent beads, and the movement of these beads was tracked using a custom Matlab script based on published algorithms[6] as well as the SpotTracker plug-in in ImageJ (NIH) in order to determine the deflection of the micropillars. The force on a microtissue during magnetic actuation was determined from the deflection of the non-magnetic pillar. For small deflections, the pillars had a spring constant k = 0.59 µN/µm. This value was determined empirically and through analytic beam bending theory[3]. Finite element modeling was used to determine the force-deflection curves for larger, non-linear deflections[7]. Tissue strain was quantified using a texture correlation image analysis algorithm written in Matlab applied to the central region of the tissues and from the micropillars' deflections[1,8].

Flexible mPAD Devices: Fabrication, Seeding and Application of Strain to Single Cells. Flexible PDMS membranes were fabricated by allowing 0.5 mL of uncured 1:20 primer:base ratio PDMS to fill the wells of a standard 6-well cell culture plate. Once cured, the membranes were removed and cut to 3 cm diameter, resulting in a membrane approximately 300 µm thick. Negative molds for micropost array devices (mPADs) were filled with PDMS by placing a single drop of uncured 1:10 PDMS between two molds[9]. Each individual mold was then cleaned of excess PDMS and placed face down onto a pre-cured flexible PDMS membrane. During subsequent curing, the post arrays fused with the membrane and remined on the membrane's surface when the mold was removed. The micropost arrays consisted of 1.8 µm diameter posts, 5.7 µm in height, arranged in hexagonal close-packed arrays, with lattice constant 4 µm. The effective spring constant for the microposts for small deflections was 22 nN/µm.

To seed cells on the stretchable mPADs, the mPAD membranes were mounted in a custom stretch chamber[10] (Supplementary Fig. 6) using sterile vacuum grease (Corning) to form a seal and contain media. SMCs were incubated for 24h on the mPADs to allow the cells to adhere and spread prior to biaxial stretch experiments. The edges of the membrane also provided a seal to a cylindrical cavity surrounding the media chamber. The application of vacuum with an automated syringe pump to this cavity produced biaxial strain on the mPADs.

Comparison of Model to Data. The materials parameters for each microtissue were obtained by fitting the incremental stress vs. time curves to the experimental results using a random search algorithm[11], where the incremental strain vs. time curves are known. For each group of parameters, the simulation curves were generated and compared with the experimental data. The optimized parameters were obtained by minimizing the normalized sum of squared residuals χ^2 until $\chi^2 < 2.5\times10^{-3}$ or $|\chi_{k+1}^2 - \chi_k^2|/\chi_k^2 < 0.001$, where k is the number of iteration. The normalized sum of squared residuals was defined as the difference between the simulation stress, $\sigma(t_i)$, and the experimental stress, $\sigma_e(t_i)$,

$$\chi^2 = \frac{1}{N(\sigma_e^{max} - \sigma_e^{min})^2} \sum_{i=1}^{N} \left(\sigma(t_i) - \sigma_e(t_i)\right)^2$$

where i denotes the ith data point, N the number of data points, and σ_e^{max} and σ_e^{min} are the maximum and minimum experimental stresses, respectively.

Myosin activation. In order to enhance the active component of the tissues' mechanical response, we constitutively activated the myosin by infecting cells and tissues with an adenovirus coding for the upregulation of RhoA (RhoA-V14), following published procedures[12]. The virus solution was added to culture media for 24 h immediately following cell seeding into microtissue wells. The virus+media solution was aspirated from the devices after this 24 h incubation, after which normal media was used for the remainder of the experiments.

Supplementary Figures and Tables:

1. Time evolution of microtissue force generation

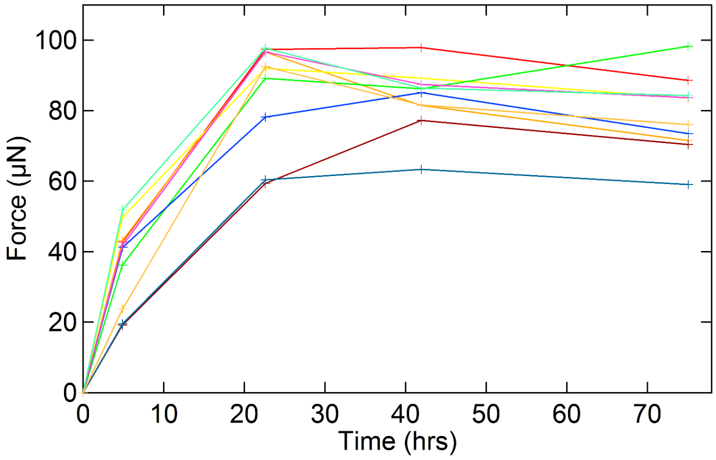

Supplementary Figure 1. Microtissue force generation over time. The force generation of selected microtissues was recorded from the moment immediately after seeding to 78 h post seeding. Microtissue force was seen to plateau by 48 hours. Linear pillar bending mechanics was used in this analysis as we were only concerned about the relative, and not absolute, changes in force generation.

2. Baseline stress and initial stiffness of microtissues

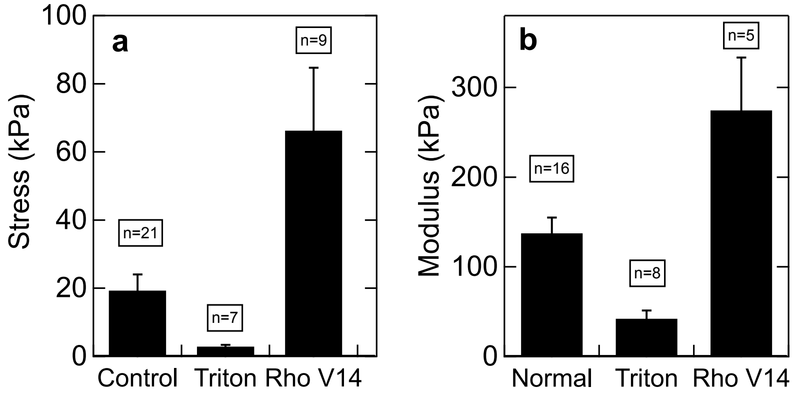

Supplementary Figure 2. Microtissue baseline stress and initial stiffness. (**a**) Microtissue contractility is strongly influenced by myosin activity. Baseline average stress for microtissues prior to mechanical actuation measurements. Control conditions: 48 hours incubation after seeding. Triton: 48 hours incubation, followed by cell lysis in 0.1% Triton X-100. Rho V14: 48 hours incubation, with adenovirus encoding constituitively active Rho to upregulate myosin activity added to culture media during first 24 hours following seeding. (**b**) Microtissue stiffness, as measured by initial slope of stress-strain curves.

3. Additional examples of dynamic response of microtissues to magnetic force.

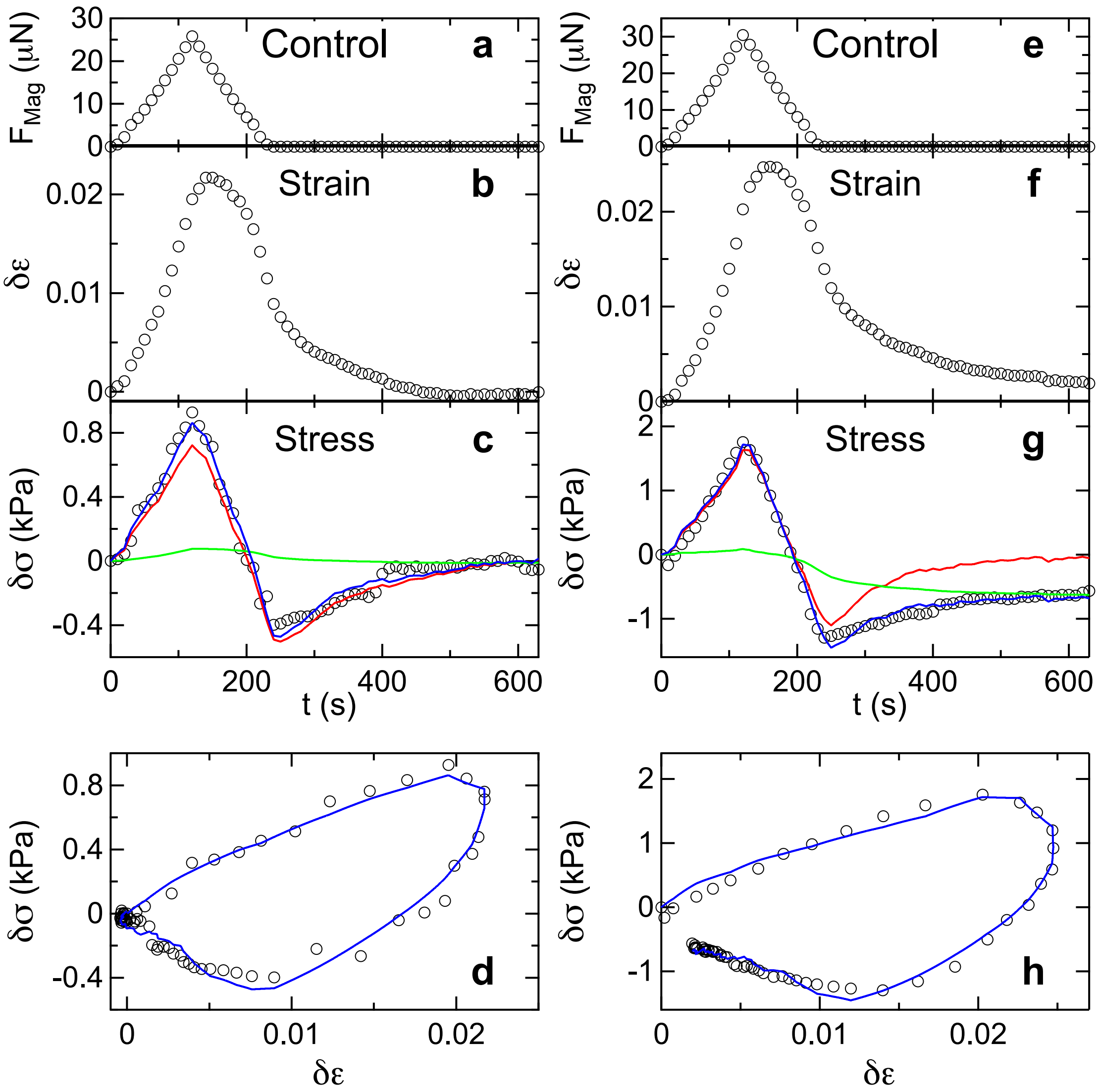

Supplementary Figure 3. Dynamic response of as-grown control microtissues. The responses of tissues C2 (left) and C3 (right) to magnetic forcing are shown. (**a**),(**e**) Magnetic force profile $F_{Mag.}$ vs time t applied via Ni sphere. (**b**), (**f**) Resulting incremental strain $\delta\varepsilon$ vs t. (**c**), (**g**) Incremental stress $\delta\sigma$ vs t. (**d**), (**h**) Incremental stress-strain curves $\delta\sigma$ vs $\delta\varepsilon$. $\delta\sigma$ and $\delta\varepsilon$ are measured relative to the as-grown tissue configurations. Baseline stresses were σ_0 = 16.7 kPa and 21.1 kPa for Tissues C2 and C3, respectively. The blue curves are the result of fits to the model described in the text and shown in Fig. 3. The red and green traces in (**c**) and (**g**) show the cell and ECM contributions to the incremental stress $\delta\sigma_a$ and $\delta\sigma_p$, respectively, as determined from the model. The parameters for these fits are given in Supplementary Table 1.

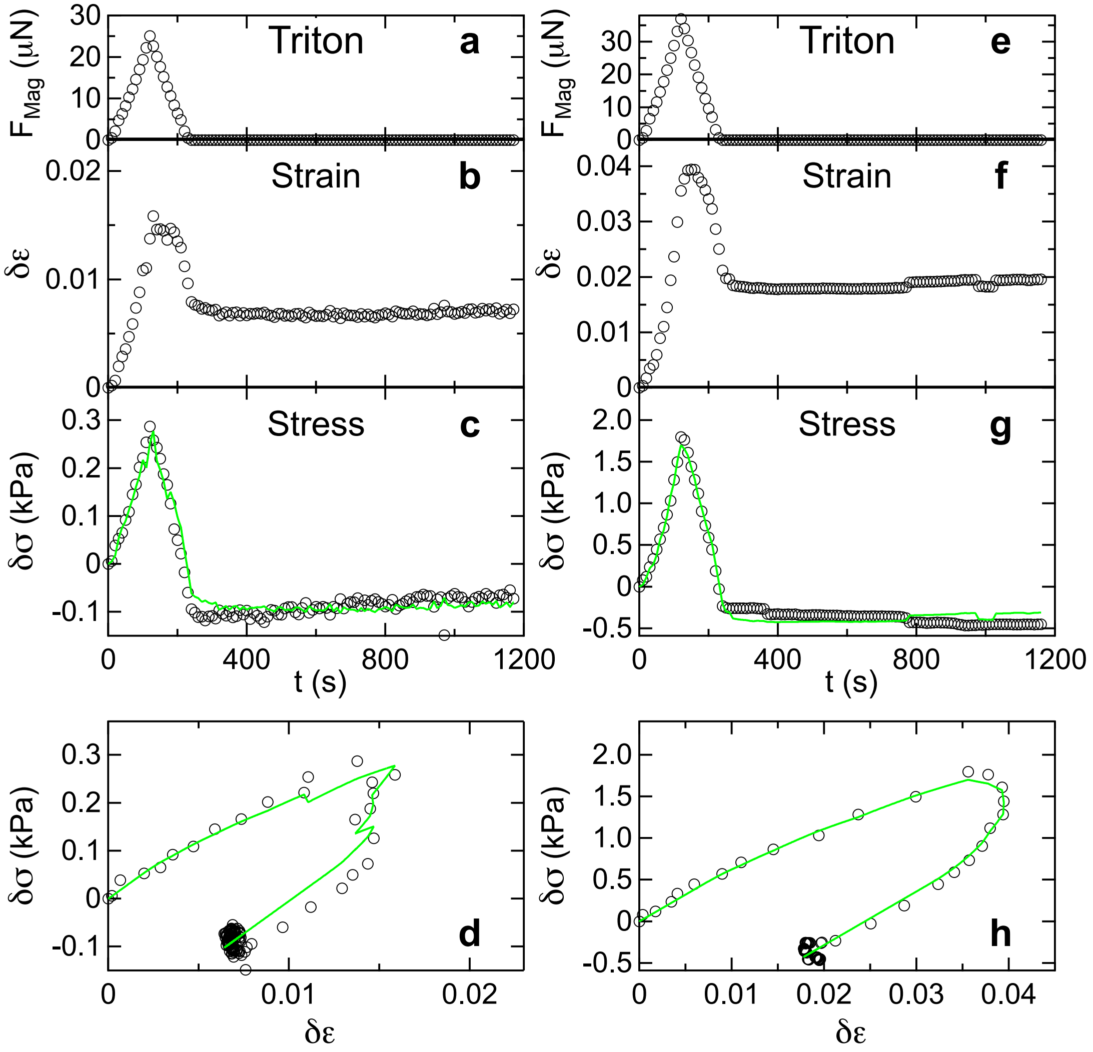

Supplementary Figure 4. Dynamic response of Triton-X 100 treated microtissues. The responses of tissues T2 (left) and T3 (right) to magnetic forcing are shown. (**a**), (**e**) Magnetic force profile $F_{Mag.}$ vs time t applied via Ni sphere. (**b**), (**f**) Resulting incremental strain $\delta\varepsilon$ vs t. (**c**), (**g**) Incremental stress $\delta\sigma$ vs t. (**d**), (**h**) Incremental stress-strain curves $\delta\sigma$ vs $\delta\varepsilon$. $\delta\sigma$ and $\delta\varepsilon$ are measured relative to the tissue configuration following Triton treatment, with baseline stresses σ_0 = 1.17 kPa and 6.7 kPa for Tissues T2 and T3, respectively. The green curves are fits encompassing ECM contributions of the model only as described in the main text. The parameters for these fits are given in Supplementary Table 1.

4. Linearity of microtissue response over experimental range of applied strains

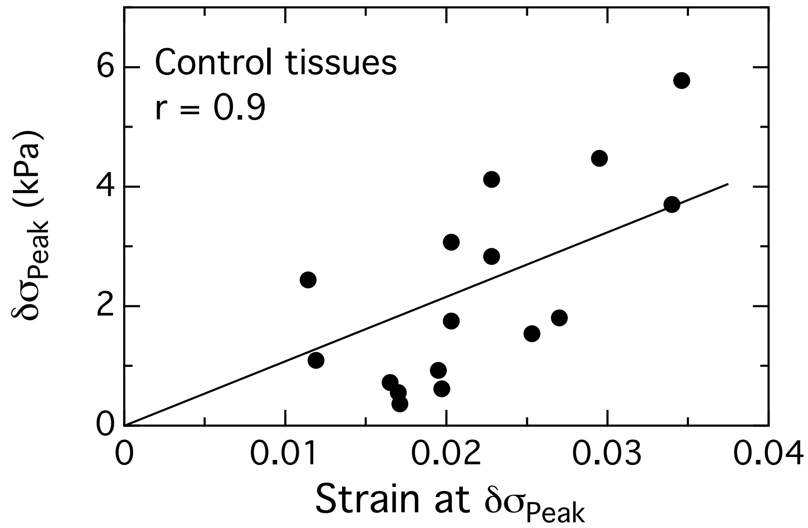

Supplementary Figure 5. Linearity of microtissue response. This figure shows the peak incremental stresses $\delta\sigma_{Peak}$ induced in a set of control microtissues vs the corresponding incremental strain at that stress. Each data point is for a different microtissue. A linear fit to these data constrained to pass through the origin (solid line) yields a correlation coefficient r = 0.9. This result provides support for the use of linear elasticity to describe the series and parallel elements in our model.

5. Tabulated results of fits to dynamic stretching data

The results of the fits to the microtissue stretching data using the model described in the text are shown in Supplementary Table 1 below. As noted in the text, for the control tissues, the modeling is largely insensitive to the viscoplastic elements of the model and also the parallel stiffness E_p, for which fits of reasonable quality encompassing a range of parameters were obtained, as illustrated in Supplementary Fig. 6 below. We also note that the model did not enable an unambiguous determination of the baseline stresses σ_a^0 and σ_p^0 for the control tissues, since we only measured their sum $\sigma_0 = \sigma_a^0 + \sigma_p^0$. In particular, since $\sigma_p^0 = \sigma_y^c$, we could not access the compressive yield stress. The fitted tensile yield stress σ_y^t is also dependent on the baseline parallel stress σ_p^0, which cannot be determined based on the available data. For Tissue C2, the fitted mechanical response of parallel element is more elastic (than viscoelastic), so we conclude that the tensile yield stress σ_y^t is beyond the current maximum parallel stress $\sigma_p^0 + 0.17$ kPa.

For the untreated control tissues, Supplementary Table 1 also shows the estimated microtissue active viscosity η_a^t based on the average single-cell active viscosity η_a^c = 0.30 ± 0.09 mN·s determined from the measurements on mPADs (Fig. 4), the initial numbers of cells per

well $N_{initial}$, the average microtissue volume V_t = 4x10^6 µm^3, and Eqn. 5, $\eta_a^t = \rho_c^{2/3}\eta_a^c$, where $\rho_c = N_{initial}/V_t$.

Supplementary Table 1: Model Fit Parameters

**Sample ID**	**Tissue type**	**Active viscosity** η_a(MPa. s)	**Series stiffness** E_s(kPa)	**Parallel stiffness** E_p(kPa)	**Tensile Yield stress** σ_y^t(kPa)	**Plastic viscosity** η_p(MPa. s)	**N initial**	**Est.** η_a^t(MPa. s)
C1	Control	1.6	30.2	0.9	$\sigma_p^0 + 0.02$	0.012	310	0.55
C2	Control	3.8	82.7	7.8	$> \sigma_p^0 + 0.17$	0	460	0.71
C3	Control	9.5	198	27.8	$\sigma_p^0 + 0.06$	0.28	410	0.65
T1	Triton-X	N/A	N/A	12.9	0.37	1.1		
T2	Triton-X	N/A	N/A	27.2	0.047	2.0		
T3	Triton-X	N/A	N/A	64.3	0.49	3.9		

6. Comparison of full model to simplified model for control tissues.

As noted in the main text, we found that, for the untreated control tissues, the fits to the full model were generally insensitive to the parameters associated with the matrix, namely the elasticity E_p, the compressive and tensile yield stresses σ_y^c and σ_y^t, and the viscosity η_p above the yield stress range. To test this more fully, we also fit the control tissues to a simplified model shown in the inset to Supplementary Fig. 6a, which neglected the viscoplastic element of the full model. An example of such a fit for tissue C1 (data also shown in Fig. 2) with E_p = 0 is shown in Supplementary Figs. 6a and 6b, where it may be seen that this fit compares quite well to the full model fit. Varying E_p in the range 0-21 kPa gave comparable results. Even in cases where some plasticity could be observed in the control tissues, such as Tissue C3 (data also shown in Supplementary Fig. 3), the simplified model does surprisingly well, as shown in Supplementary Figs. 6c and 6d.

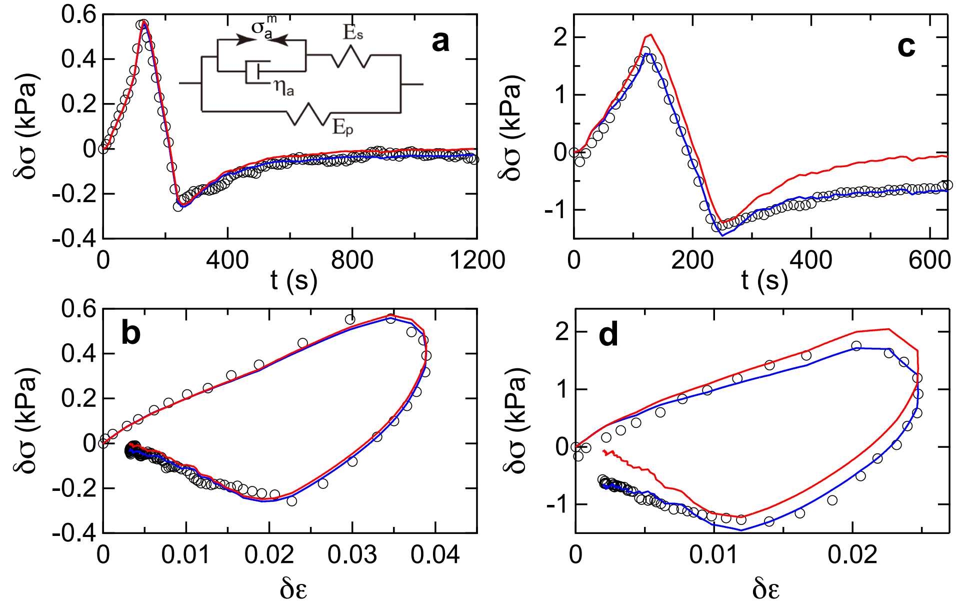

Supplementary Figure 6. Comparison of full model (Fig. 3) fits for control tissues to simplified model that includes only the active components and series passive components. (**a**), (**b**) Control tissue C1 (Fig. 2). (**c**), (**d**) Control tissue C3 (Supplementary Fig. 3). In all panels, the blue curves are the fits to the full model shown in Fig. 2 and Supplementary Fig. 3 for tissues C1 and C3, respectively. The red curves are the simplified model fits. Parameters for the simplified model fits shown are for tissue C1: η_a = 1.8 MPa·sec and E_s = 30.3 kPa; for tissue C3: η_a = 9.5 MPa·sec and E_s = 202 kPa.

7. Schematic of mPAD stretching chamber.

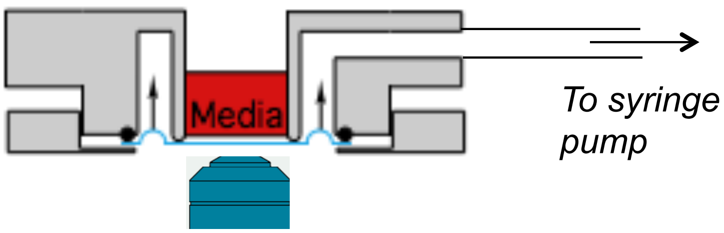

Supplementary Figure 7. Cross-sectional view of chamber for vacuum-controlled stretching of single cells on flexible micropost array detectors (mPADs). The mPAD substrate (blue) was mounted across a circular support with a grease seal to contain culture media (red). An o-ring seal (black dots) enabled application of vacuum via a programmable syringe pump to stretch the mPAD array. The system allowed continuous imaging during stretch via an inverted microscope objective (blue).

References

1 Zhao, R., Boudou, T., Wang, W. G., Chen, C. S. & Reich, D. H. Decoupling cell and matrix mechanics in engineered microtissues using magnetically actuated microcantilevers. *Adv. Mater.* **25**, 1699-1705, (2013).

2 Zhao, R., Boudou, T., Wang, W. G., Chen, C. S. & Reich, D. H. Magnetic approaches to study collective 3D cell mechanics in long-term cultures (invited). *J. Appl. Phys.* **115**, 172616, (2014).

3 Legant, W. R. *et al.* Microfabricated tissue gauges to measure and manipulate forces from 3D microtissues. *Proc. Natl. Acad. Sci. U. S. A.* **106**, 10097-10102, (2009).

4 Boudou, T. *et al.* A Microfabricated Platform to Measure and Manipulate the Mechanics of Engineered Cardiac Microtissues. *Tissue Eng. Part A* **18**, 910-919, (2012).

5 Lin, Y. C., Kramer, C. M., Chen, C. S. & Reich, D. H. Probing cellular traction forces with magnetic nanowires and microfabricated force sensor arrays. *Nanotechnology* **23**, 075101, (2012).

6 Crocker, J. C. & Grier, D. G. Methods of digital video microscopy for colloidal studies. *J. Colloid Interface Sci.* **179**, 298-310, (1996).

7 Zhao, R., Chen, C. S. & Reich, D. H. Force-driven evolution of mesoscale structure in engineered 3D microtissues and the modulation of tissue stiffening. *Biomaterials* **35**, 5056-5064, (2014).

8 Zhao, R. & Simmons, C. A. An improved texture correlation algorithm to measure substrate–cytoskeletal network strain transfer under large compressive strain. *J. Biomech.* **45**, 76-82, (2012).

9 Fu, J. P. *et al.* Mechanical regulation of cell function with geometrically modulated elastomeric substrates. *Nat. Methods* **7**, 733-736, (2010).

10 Copeland, C. R. *Cellular forces and mechanical coupling using microengineered devices* Ph.D. thesis, Johns Hopkins University, (2014).

11 Bergstra, J. & Bengio, Y. Random Search for Hyper-Parameter Optimization. *Journal of Machine Learning Research* **13**, 281-305, (2012).

12 McBeath, R., Pirone, D. M., Nelson, C. M., Bhadriraju, K. & Chen, C. S. Cell shape, cytoskeletal tension, and RhoA regulate stem cell lineage commitment. *Developmental Cell* **6**, 483-495, (2004).
